# Temporal profiles for measuring threshold of random lasers pumped by ns pulses

**DOI:** 10.1038/s41598-017-05513-8

**Published:** 2017-07-13

**Authors:** Xiaoyu Shi, Qing Chang, Junhua Tong, Yunjie Feng, Zhaona Wang, Dahe Liu

**Affiliations:** Applied Optics Beijing Area Major Laboratory, Department of Physics, Beijing Normal University, Beijing, 100875 China

## Abstract

The working threshold is an important parameter to assess the performance of cavity-free random lasers. Here, the temporal profile measurement is proposed as an alternative method to determine the thresholds of the surface plasmon based random lasers pumped by ns pulses based on analyzing the delay time (*t*_Delay_) and rising time (*t*_R_) of the emission signal. The obvious and slight inflection points of the curves of *t*_Delay_ and *t*_R_ varying with the pump power density are observed as indicators for the thresholds of random lasing and for the transition of lasing mode, respectively. The proposed method supplies consistent values to those supplied by traditional methods in frequency-domain for the random systems with different gain length. The demonstrated temporal profile approaches are free from the spectrometers and may be as a candidate for measuring the threshold of random lasers in ultrafast optics, nonlinear optics and bio-compatible optoelectronic probes.

## Introduction

Random lasers (RLs)^1–5^ based on both the dielectric scattering^6–10^ and the surface plasmonic resonance of metallic nanoparticles^11–17^ have attracted wide attentions for their interesting physical mechanism and potential value in applications such as light sources^18^, speckle-free imaging^19^, and information retrieval^20^. For RLs, the working threshold is one of the most important physical parameters for characterizing their performances. Generally, the variation inflection point of the emission intensity and/or spectral linewidths with the pumping power density has been used to determine the threshold for random lasing^11–17^. The probability of random lasing in the case of random lasers^21^ with sub-nanometric spikes on the emission curves, and the Lêvy exponents^22^ are also used to define the threshold, respectively. These methods mentioned above are based on the emission spectra in the frequency domain, and a spectrometer is needed. Moreover, the inherent random characteristic of random lasing means intensely fluctuations of emission spectra in terms of intensity, bandwidth and spike locations, especially around the threshold^21, 23–25^. The fluctuation^23, 24^ property makes the process of measuring the random lasing threshold based on the spectra in the frequency domain is complicated. Therefore, an alternative method to determine thresholds is in great need to present for random lasers even when there is no spectrometer.

Fortunately, the temporal profile of the emission contains some valuable kinetics of relaxation from the excited to the ground state^25^, such as the lifetime of the radiative recombination of the excitons^26–28^ and the optical wave-matter interaction processes: linear fluorescence^25^ and nonlinear stimulated emission^29, 30^. For RLs, temporal profile measurements have been widely used to demonstrate the dynamic response of RLs and the decay time variation with the pumping power density^29–33^. Particularly, the transition in temporal and spectral features of emission was theoretically and experimentally demonstrated at the same pump energies (the threshold) in a previous report^29, 30, 34^. However, there are no reports on tracking the thresholds of RLs based on the delay time relative to the pumping pulse and the rising time in the temporal profile of the emission curves.

In this work, an alternative method is presented for determining the thresholds of surface plasmon based random lasers pumped by ns pulses based on analyzing the delay time (*t*_Delay_) and rising time (*t*_R_) of the temporal profiles varying with the pump power densities. Dynamic behaviors of the emission from the four dye-based RL systems are studied by using a detector and an oscilloscope. The evolutions of delay time relative to the pumping pulse and the rising time show a universal change rule versus pump power densities, demonstrating a obvious inflection point corresponding to the threshold of random lasing measured by traditional methods in frequency-domain. More importantly, a slight inflection is also observed to demonstrate the transition of random lasing modes. The proposed temporal profile approaches may be act as a candidate for measuring the threshold of dye random lasers in the ultra-fast optical field.

## Theoretical Analysis

**Figure 1:**
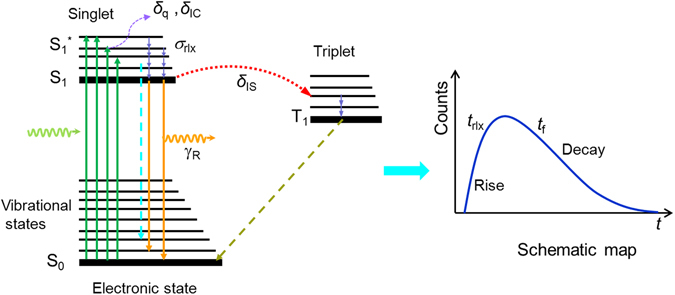
Working mechanism of temporal profile based threshold measurement. Photo-physics processes in a simplified energy level diagram of an organic molecule R6G (left panel) and pictorial representation of the fluorescence intensity with time (right panel). The full lines represent the electronic states whereas the thin lines represent the vibrational states.

When a pumping beam is incident on a random system with gain materials, some of the photons are very rapidly (10−15 fs) absorbed. The relevant photo-physics is shown in Fig. 1 ^25^. First, a Rhodamine 6 G (R6G) molecule is excited to a vibrational level S_1_* of the excited electronic states by a nanosecond laser pulse. Most molecules relax from the excited state S_1_* to the lowest level of the singlet state S_1_ through vibrational relaxation at a rate of *σ*_rlx_. Then, the molecule populating the state S_1_ can de-excite itself to the ground level through a radiative process at a rate of *γ*_R_ and a non-radiative decay process at a rate of *γ*_NR_, which involves various other non-emission channels such as an internal conversion process with a decay rate of *δ*_IC_, intersystem crossing with a decay rate of *δ*_IS_, and a quenching process with a decay rate of *δ*_q._ The non-radiative rate and the fluorescence rate are given by the formula *γ*_NR_ = *δ*_IC_ + *δ*_IS_ + *δ*_q_ and *γ*_F_ = *γ*_R_ + *γ*_NR_, respectively. Other photons of the incidental beam are scattered and subsequently absorbed by R6G molecules as analyzed above or escape from the random system. At the same time, few excited molecules can directly transits from vibrational level S_1_* to the ground level through a Raman scatter based radiative process^35^. This process may shorten the pulse duration by decreasing the rising time of emission induced by the leaving out of *σ*_rlx_. Based on the transition processes mentioned above, the non-radiative relaxation from the photo-excited state S_1_* to the emitting state S_1_ is measured through the rising time of the emission temporal profile^25^. The non-radiative relaxation from the emitting state S_1_ to the ground state S_0_ can be studied through the falling time, which reflects the lifetime *t*_f_. Based on this physical mechanism, the temporal profile measurement technique is used to study the dynamic behaviors of RLs and analyze their threshold behaviors.

### Experimental design

**Figure 2:**
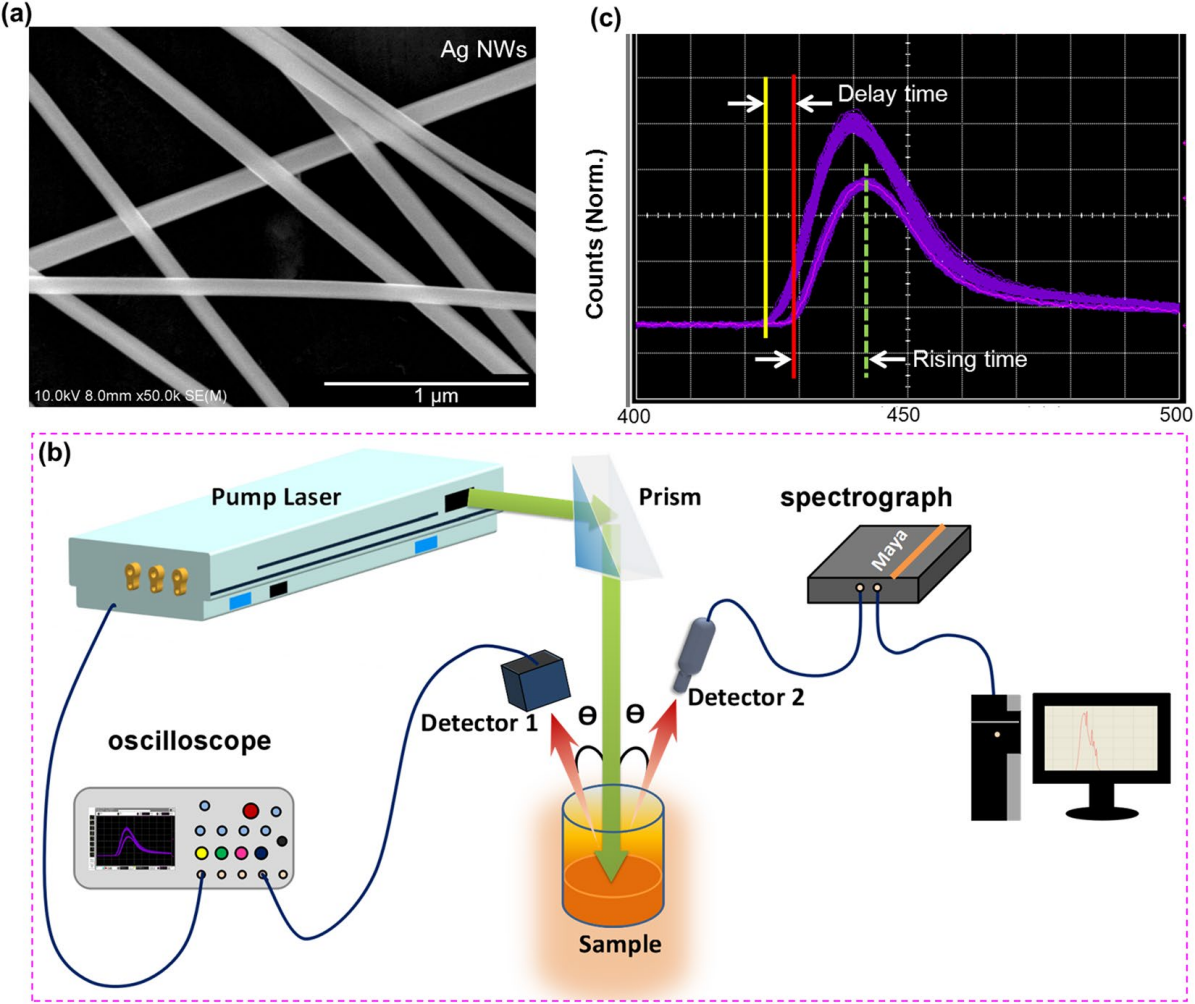
Experimental configuration. (**a**) The scanning electron microscope (SEM) image of Ag NWs. (**b**) The experimental setup. The Nd:YAG ns pulses with a wavelength of 532 nm are reflected by a prism, and then normally pump the random systems in a beaker. The frequency-domain emission spectra are recorded by using an optical fiber spectrometer. Simultaneously, the temporal profile signal is recorded by a fast-response photoelectric detector and displayed by an oscilloscope. (**c**) The screen picture of the temporal profile spectrum of RL emissions from an oscilloscope and the diagram defining the delay time relative to the pumping pulse and rise time.

In our experiment, silver nanowires (Ag NWs) are synthesized by a polyvinylpyrrolidone-assisted reaction in ethylene glycol^11, 36^. The scanning electron microscope (SEM) image of the Ag NWs in Fig. 2a shows that the diameter of the Ag NW is 110 ± 20 nm with a length of up to 20–50 μm. Dispersing the Ag NWs into an ethanol solution of R6G at a concentration of 2.1 mM, a random system is obtained with an Ag NW concentration of 0.88 mg/mL (ρ = 7.3 × 10^7^ mL^−1^). The corresponding mean free path of the system is estimated to be l* > 1.9 cm by using the transmission spectra in the range of 530–560 nm^33^, which can be treated as a weakly scattering system^12–18^. The pumping source is a frequency-doubled Nd:YAG laser (Continuum Model PowerLite Precision 8000) with a wavelength of 532 nm, a pulse duration of 10 ns, an output beam diameter of 8 mm, and a repetition rate of 10 Hz. The pump beam reflected by a prism is normally incident on the random scattering dispersion in a beaker with a diameter of 40 mm and a height of 50 mm, as shown in Fig. 2b. The emission spectrum is recorded by an optical fiber spectrometer (Ocean Optics model Maya Pro 2000) with a spectral resolution of 0.4 nm. Meanwhile, the emission is filtered by a high pass filter with cut-off wavelength 540 nm and then the temporal signal of emission is measured by a detector (Electro-Optics Technology model ET 2030) with the rise time of 300 ps and an oscilloscope (Agilent model 54832B) with the bandwidth of 1 GHz. Thus the time between the starting point and the highest point of the temporal profile is defined as the rise time *t*_R_ as shown in Fig. 2c. The detecting angle is about 10° with respect to the pumping beam to avoid reabsorption (labeled as θ in Fig. 2b). To reveal the effective interplaying time between the pulse and the random system, the pulse reflected by a paper replacing the random system is recorded and shown on the screen of oscilloscope. And the time interval of the pulse relative to the internal trigger point of pulsed laser is obtained. By the same way, the other time interval of the emission light signal relative to the internal trigger point is also measured. Through the two-step measurement, the delay time between the pump source (purple line) and emission signal from the random system (pink line) can be measured and recorded as the delay time *t*_delay_.

## Results and Discussions

**Figure 3:**
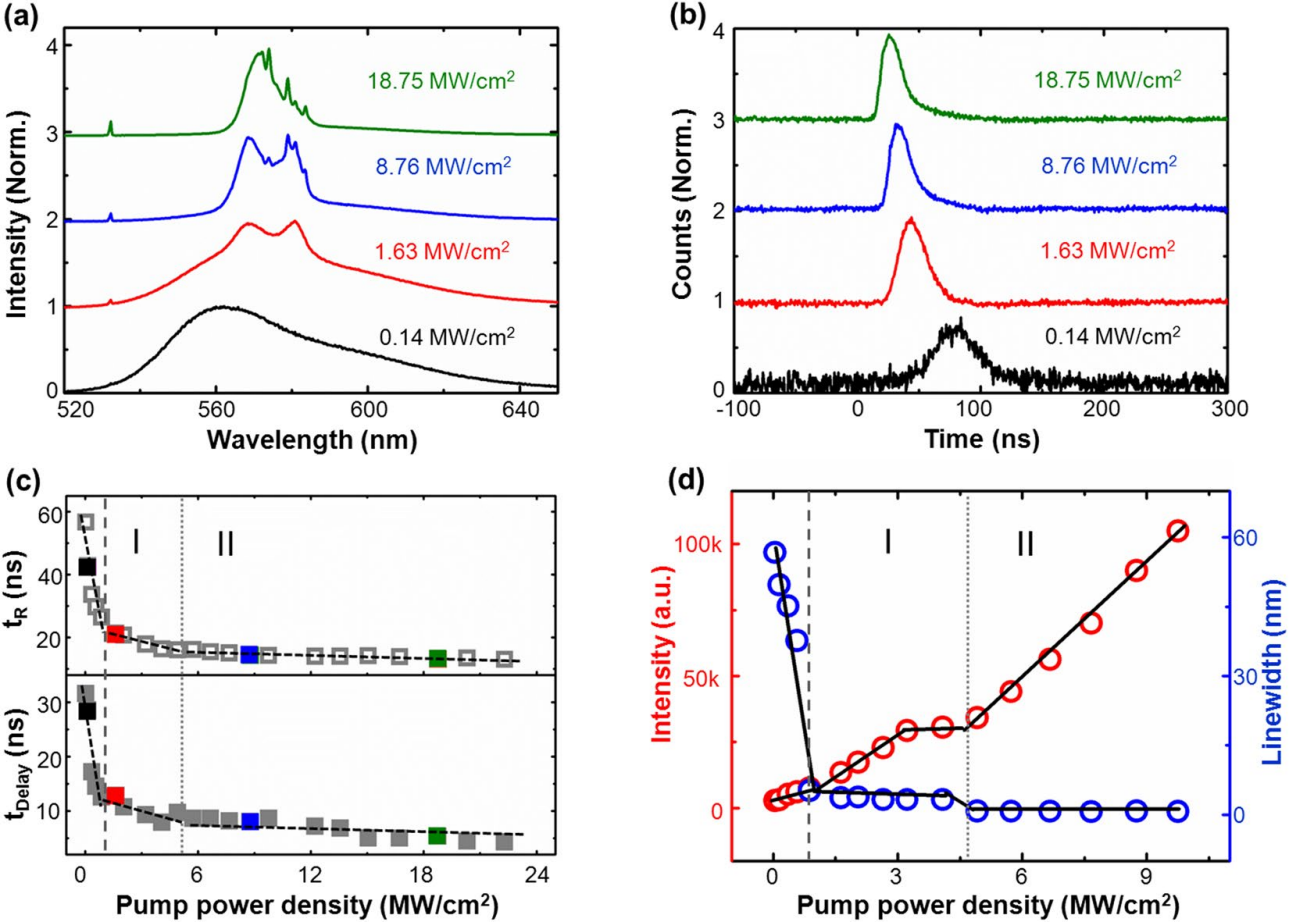
Transition in spectral and temporal features of emission. (**a,b)** The frequency-domain spectrum (**a**) and the temporal profile signal (**b**) from the Ag-nanowire-based dual-regime RL with R6G in a concentration of *C*_R6G_ = 2.1 mM under different pump power densities of 0.14 MW/cm^2^ (black line), 1.63 MW/cm^2^ (red line), 8.76 MW/cm^2^ (blue line), and 18.75 MW/cm^2^ (olive line). (**c**) Variation of delay time (bottom) and rise time (top) with pump power density. Four colored data points coordinate with the selected data in **a** and **b** while other points are gray. (**d**) The intensity and bandwidth of emission at 573.99 nm change with the pump power density.

A typical dual-regime random laser^36^ is chosen and its emission characteristics are demonstrated in Fig. 3 under the condition of *C*_R6G_ = 2.1 mM with *ρ*_AgNW_ = 7.3 × 10^7^ mL^−1^. The emission spectra (Fig. 3a) and the temporal profiles (Fig. 3b) are plotted under different pump power densities of 0.14 MW/cm^2^ (black line), 1.63 MW/cm^2^ (red line), 8.76 MW/cm^2^ (blue line), and 18.75 MW/cm^2^ (olive line). It can be seen that, under the low pump power density of 0.14 MW/cm^2^, there is only broad photoluminescence with an emission linewidth of 55 nm. From the corresponding temporal profile of photoluminescence, the delay time with respect to the pumping pulse is 28.6 ns and the rising time is 42.83 ns in black in Fig. 3b. By increasing the pump power density to 1.63 MW/cm^2^, the emission linewidth decreases to 7 nm, implying that random lasing^2^ is built up in the system. Correspondingly, the delay time and the rising time decrease to 12.86 ns and 21.33 ns obtained from the red curve in Fig. 3b, respectively. After increasing the pumping power density to surpass 4.59 MW/cm^2^, the emission spectrum begins to contain several discrete sharp spikes with linewidth less than 1 nm in region II, which is the distinctive characteristic of random lasing and reveals one transition point of random lasing modes. It should be pointed out that these random lasing peaks are located at 573.77 nm, 578.79 nm, 580.84 nm, 583.49 nm, which are in accordance with the Raman shift of R6G molecules at 1361 cm^−1^, 1509 cm^−1^, 1575 cm^−1^, 1651 cm^−1^ ^37^. This high concordance shows the discrete of random lasing modes related to the Raman scattering process^38, 39^. And the transition point of random lasing modes may be induced by nonlinear effect^40^ and self-phase modulation^41^. Meanwhile, the delay time and the rise time decrease to 8.07 ns and 14.25 ns, respectively. When the pump power density is further increased, these distinct peaks become higher, accompanied with the delay time of 5.07 ns and rising time of 13.07 ns which is almost unchanged.

To demonstrate the threshold behavior of this random system, Fig. 3c presents the evolution of the rise time *t*_R_ and the delay time *t*_Delay_ with increasing the pump power densities (more information seen the Supplementary Fig. S1). It shows one obvious inflection point at 0.96 MW/cm^2^ correspond to the working threshold for random lasing and one slight inflection at 4.57 MW/cm^2^ representing the random lasing transition from smooth mode to discrete mode. When the pump power density is smaller than the lasing threshold, the delay time and the rising time decrease super-linearly with increasing the pump power density. As the pump power density exceeds the threshold, the rising time and the delay time decrease more slowly. After the pump power density surpasses the lasing mode transition point from region I to region II, the variations of rising time and the delay time subside significantly. That is to say, with increasing the pump power density, the delay and rising time first shorten rapidly during the process of fluorescence, then shorten slowly owing to the lasing resonance process, and finally almost remain a constant when the distinct spikes appear in the emission curves (region II). For comparison, the threshold behavior of the RL using the traditional frequency-domain methods is plotted in Fig. 3d. The results obtained from the temporal profile measurements (lasing threshold of 0.98 MW/cm^2^ and the lasing mode transition point of 4.62 MW/cm^2^) are in good agreement with those measured by using the traditional frequency-domain method.

**Figure 4:**
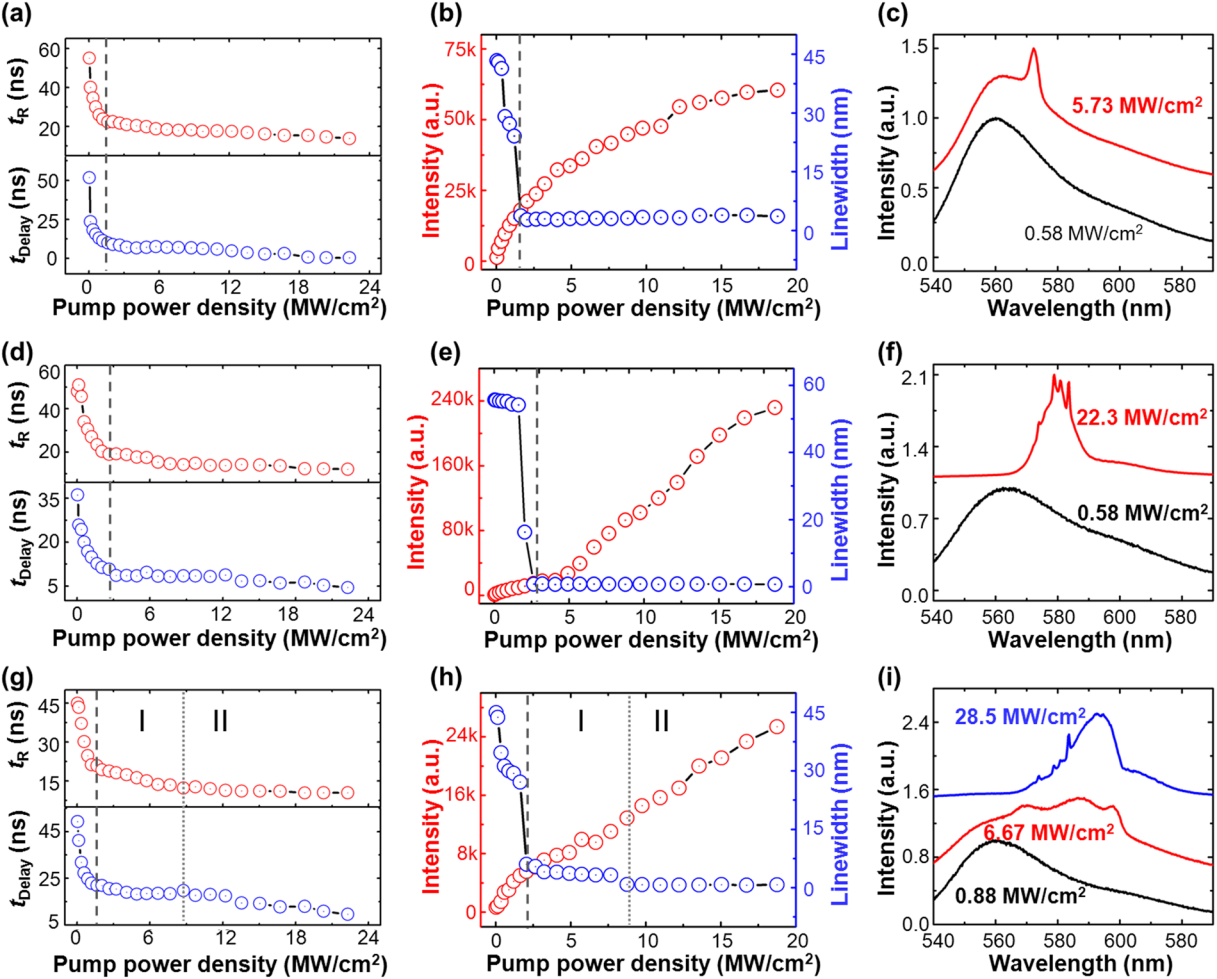
Threshold features of delay time and rise time in different random systems. Plots are the results from three systems, S2, S3, and S4, with varying *C*_R6G_ concentrations of 0.1 mM, 10.4 mM, and 18.8 mM, respectively. (**a**,**d**,**g**) The variation of delay time (bottom) and rise time (top) versus pump power density of S2 (**a**), S3 (**d**), and S4 (**g**); (**b**,**e**,**h**) The variation of intensity and linewidth versus pump power density of S2 (**b**), S3 (**e**), and S4 (**h**); (**c**,**f**,**i**) The normalized frequency domain spectrum of S2 (**c**), S3 (**f**), and S4 (**i**).

The temporal profile of the three additional random systems based on three different R6G concentrations with a fixed concentration of Ag NWs (7.3 × 10^7^ mL^−1^) are also studied and shown in Fig. 4 as an identifier for their thresholds. The random system mentioned above with the concentration of R6G as 2.1 mM is called S1, while a concentration of 0.1 mM, 10.4 mM, and 18.4 mM, are labeled as S2, S3, and S4, respectively. Figure 4a,d and g show the variation of the rising time and the delay time versus pump power densities for systems S2, S3, and S4, respectively (more information seen the Supplementary Fig. S2). According to the evolution regular of the curves, the thresholds of three systems are presented in accordance with the results measured by the traditional intensity-based or linewidth-based method (shown in Fig. 4b,e and h). The lasing threshold for S2 is 1.37 MW/cm^2^. There is no discrete random lasing modes in the emission curves shown in Fig. 4c with a fluorescence spectrum at 0.58 MW/cm^2^ and a narrowing lasing spectrum at 5.73 MW/cm^2^. For the system of S3, random lasing is formed at the threshold of 2.71 MW/cm^2^. The corresponding spectra are plotted in Fig. 4f, showing the fluorescence spectrum at 0.14 MW/cm^2^ and the discrete random lasing modes at 22.29 MW/cm^2^. In Fig. 4g, the curve contains two inflection points, representing the lasing threshold of 1.61 MW/cm^2^ and the random lasing mode transition point of 8.70 MW/cm^2^, respectively. The corresponding spectrum clearly demonstrates fluorescence behavior at 0.88 MW/cm^2^, smooth random lasing spectrum at 6.67 MW/cm^2^, and the emission curve with discrete random lasing modes at 28.5 MW/cm^2^.

The thresholds of the above four random systems (S1, S2, S3 and S4) measured by the temporal profile method (new) and frequency domain method (traditional) are presented in Table 1. The good agreement of these values indicates that the temporal profile method can be used to access the thresholds of the dye based random lasers with different gain lengths.

**Table 1:** The threshold of these four random systems (S1, S2, S3, and S4), measured by the temporal profile method (new) and frequency domain method (traditional), respectively.

**Sample**	***E***_**th**_ **(MW/cm** ^**2**^ **)**	
	New	Traditional
**S1**	0.95	0.98
**S2**	1.36	1.47
**S3**	2.71	2.83
**S4**	1.61	1.94

It should be noted that the delay time is defined by the time interval between the pumping pulse and emission lasing pulse, which reflects the build-up time of the random emission from the stimulating pump pulse. When the pumping power density is much smaller than the threshold, there are no laser-induced nonlinear effects in such a random system, indicating the relative large refraction index and the relative uniform photonic environment. And there exists large losses in the random systems and very small optical gain. Then, the probability of a photon generating a second photon before leaving the gain regime approaches zero^42^. The emitted photons from R6G need to experience a longer path and take a longer time to obtain the guaranteed gain. Thus, a larger delay time and only weak fluorescence of R6G can be observed under the low pumping power density. When the pump energy is increased to higher value, more molecules are excited to a high level and the probability of stimulated emission is increased. Simultaneously, strong laser field generates the reduction of the environment refraction index in the pump area by the nonlinear effect^43–45^, indicating a lager transport speed of photon. And the laser-induced refractive index gradient is induced, meaning an effective poor cavity to further decrease optical path and the loss (Detailed information shown in Supporting Information)^46–48^. As a result, lasing resonance can be built-up in a shorter time before photons leave the gain regime^42^, resulting in narrowing of the linewidth and more intense emission and quickly reduction of the delay time. After the pump power reaching the lasing threshold, the gain is larger than the loss from the random system and lasing resonance is formed based on the coupling role of several nonlinear optical effects (saturation absorption^49, 50^, large refraction index gradient for optical cavity^51, 52^, stimulated Raman scattering^38, 40^, self-phase modulation^43, 53–57^, *et al*.), which may induce a relative stable delay time (more detailed information seen in the Supplementary Information). In particular, self-phase modulation process in laser cavities can shorten the pulses and improve mode-locking stability (more detailed information seen in the Supplementary Information)^43, 53–57^. The stimulated Raman scattering process may play a role of seed at several wavelengths, which attributes to the random lasing with several spikes, meaning a stable delay time.

The rising time is inversely proportional to the non-radiative relaxation rate σ_rlx_ from the photo-excited state S_1_* to the emitting state S_1_, which reflects the increasing speed of population in the S_1_ state. The short rising time means the molecule decays quickly to the emitting state and population inversion can be achieved in a short time. That is to say, the rising time represents the time to achieve population inversion in random systems. Thus, the variation of the rising time with the pump power density is similar to the behavior of the delay time.

The threshold identification method in this work is studied in a dye based random system within the ns regime based on the experimental condition of ns pulses pumping. However, we believe the temporal profile measurement of thresholds might also be applicable in picosecond random laser systems because the similar phenomenon for the temporal features of emission has been demonstrated in picosecond regime^30, 32^.

## Conclusions

As a conclusion, for the first time to our knowledge, an alternative method for measuring the working thresholds of dye random lasers pumped by ns pulses is proposed based on calculating the delay time relative to the pumping pulse and the rising time in the temporal profile of the emission at different pump power densities. The possible mechanism based on an energy level diagram is analyzed. Through comparison with the results obtained from different measurement methods, the proposed method is verified in good agreement with the traditional method in frequency domain for the random systems with different gain lengths. This new approach might be used practically to determine the threshold of random lasers and look into the dynamic characteristics of the light-matter interaction in the ultra-fast optical field.

## Electronic supplementary material

Supplementary Information
