# Supplementary material for: Temporal profiles for measuring threshold of random lasers pumped by ns pulses
Source: Sci Rep. 2017 Jul 13;7:5325. doi: 10.1038/s41598-017-05513-8 (PMC5509694; doi:10.1038/s41598-017-05513-8)
Supplement: Supplementary file 1 — Supplementary Information [file 41598_2017_5513_MOESM1_ESM.doc]

**Supplementary Information**

**Temporal profiles for measuring threshold of random laser pumped by ns pulses**

**Xiaoyu Shi, Qing Chang, Junhua Tong, Yunjie Feng, Zhaona Wang* and Dahe Liu***

*Applied Optics Beijing Area Major Laboratory, Department of Physics, Beijing Normal University, Beijing 100875, China.*

*^*^* [*zhnwang@bnu.edu.cn*](mailto:zhnwang@bnu.edu.cn) *and* [*dhliu@bnu.edu.cn*](mailto:dhliu@bnu.edu.cn)

**A: Threshold behavior of random lasers in time domain**

**B: Nonlinear optical effects in random system**

**A: Threshold behavior in temporal domain**


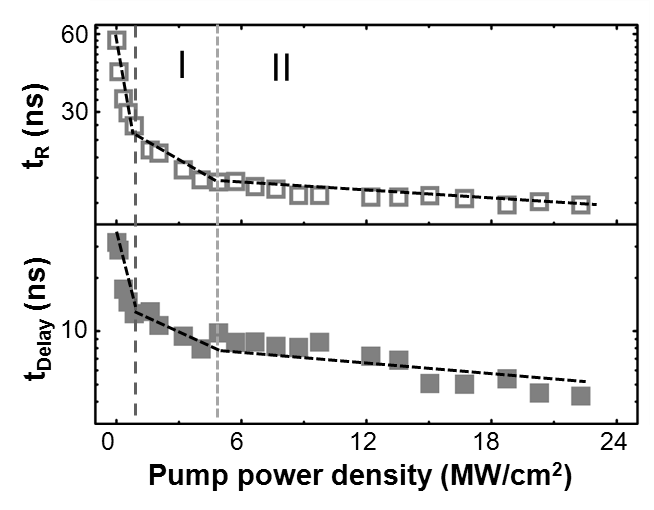


**Supporting Fig. S1** Variation of delay time (bottom) and rise time (top) with pump power density in logarithmic coordinates for the sample of S1.

Variation of delay time (bottom) and rise time (top) with pump power density in logarithmic coordinates are shown in Fig. S1 (for the sample of S1) and Fig. S2 (for the sample of S2, S3, S4), clearly exhibiting one obvious inflection point as the indicator of random lasing threshold.


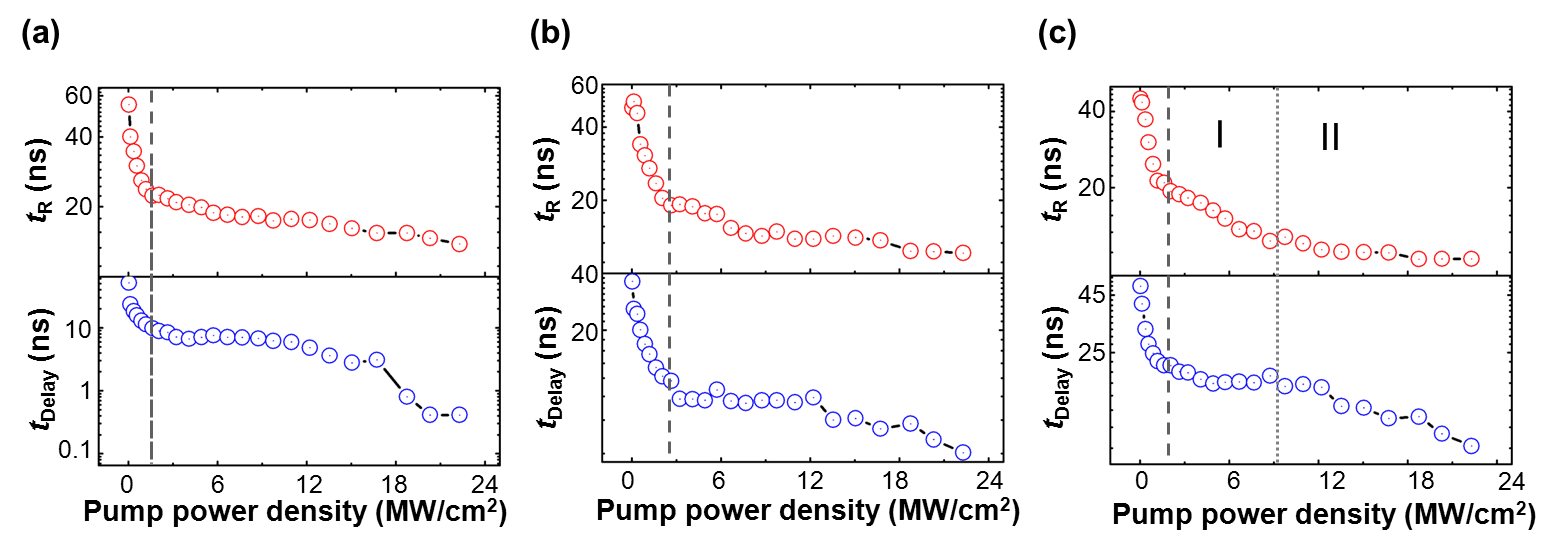


**Supporting Fig. S2** Variation of delay time (bottom) and rise time (top) with pump power density in logarithmic coordinates for the sample of S2 (a), the sample of S3 (b), and the sample of S4(c).

**B: Nonlinear optical effects in random system**

When the pump light is enough strong, several nonlinear optical effects will be induced in the Ag nanowire based random systems.

One is the laser-induced refractive index variation of the medium by photothermal effect[^1-6^](#_ENREF_1). And the distribution of refractive index is in accordance with the distribution of lasing intensity in the illuminated area,

$\Delta n(\vec{r})=nI(\vec{r})$. (1)

Considering the absorption of R6G, the isocandela lines of Gauss beam in the medium are shown in Fig. S3. And a similar distribution of the laser-induced refractive index gradient^8-10^ is induced in the illuminated area, meaning an effective poor cavity is formed in such random system.


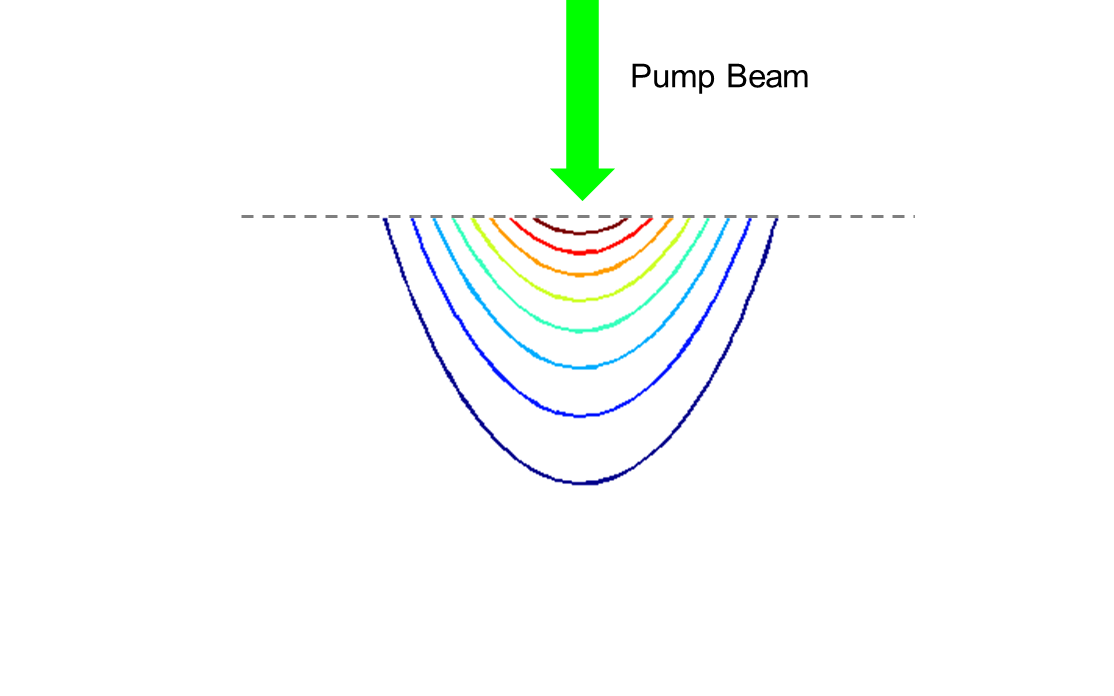


**Supporting Fig. S3** The distribution of intensity in the nonlinear material illuminated by a strong Gauss laser pulse.

The corresponding experiments were designed to illustrate the laser-induced refractive index gradient. As experimental set-up shown in Fig. S4a, the random system (Ag NW@R6G) is vertically pumped by a pulsed laser running at 532 nm (Continuum model PowerLite Precision 8000). The pulse duration was 8 ns, the repetition rate 10 Hz, and the out-beam diameter was 8 mm. Another laser beam from a He-Ne laser (632 nm) is horizontally through the pump area and light on a screen. At a low pump power density of 0.86 MW/cm^2^, the refractive index in the pump area is relative uniform and the transmission laser spot is round (in Fig. S4b). When the pump power density in increase to 2.75 MW/cm^2^, the laser spot moves down and is deformed and changed to ellipse (in Fig. S4b), indicating that the refractive index in the pump area is reduced. By further increasing the pump power density (from 6.67 MW/cm^2^ to 10.8 MW/cm^2^), the refractive index gradient becomes larger. So the deformation of laser spot is more severe (in Fig. S4b).


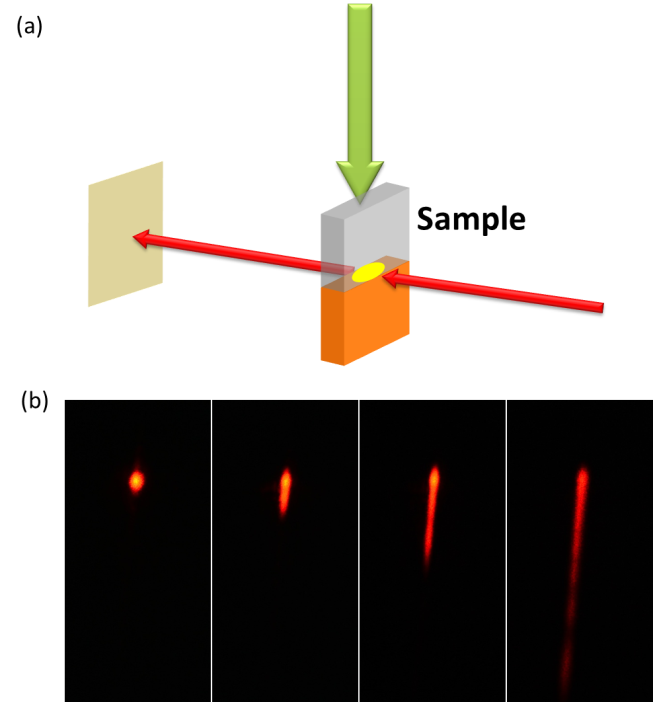


**Supporting Fig. S4** (a) Experimental set-up geometry. (b) The shape of laser spot under different pump power densities.

Another nonlinear optical effect is the spatial self-phase modulation caused by the nonlinear interplay of the intensity dependent refractive index and divergence of the propagating beam,[^8^](#_ENREF_8) charactering a pattern of diffraction rings in the far-field observed in dye solutions[^6^](#_ENREF_6) and nanoparticle dispersion[^3^](#_ENREF_3).

As shown in Fig. S5a, a CW laser (at 532 nm) with Gaussian beam is focused onto the sample by a lens of focal length 58.9 mm. The path length of Ag nanowire solution in cuvette is 10 mm. The sample with a concentration of 0.88 mg/mL is placed after the focus point (65 mm apart from the lens). After passed through the sample, the beam began to diverge into the nested array of diffraction rings, which illuminated the screen. Fig. S5b shows the variation of the diffraction ring patterns under the different pump powers of 0.05W, 0.13W, 0.2W and 0.3W, respectively. When the energy of the incidence laser exceeds 0.04W, the diffraction ring of spatial self-phase modulation is emerged. The number of rings of the diffraction ring patterns is increased by increasing the laser power. The diffraction ring pattern demonstrates spatial self-phase modulation can be induced in our random systems.


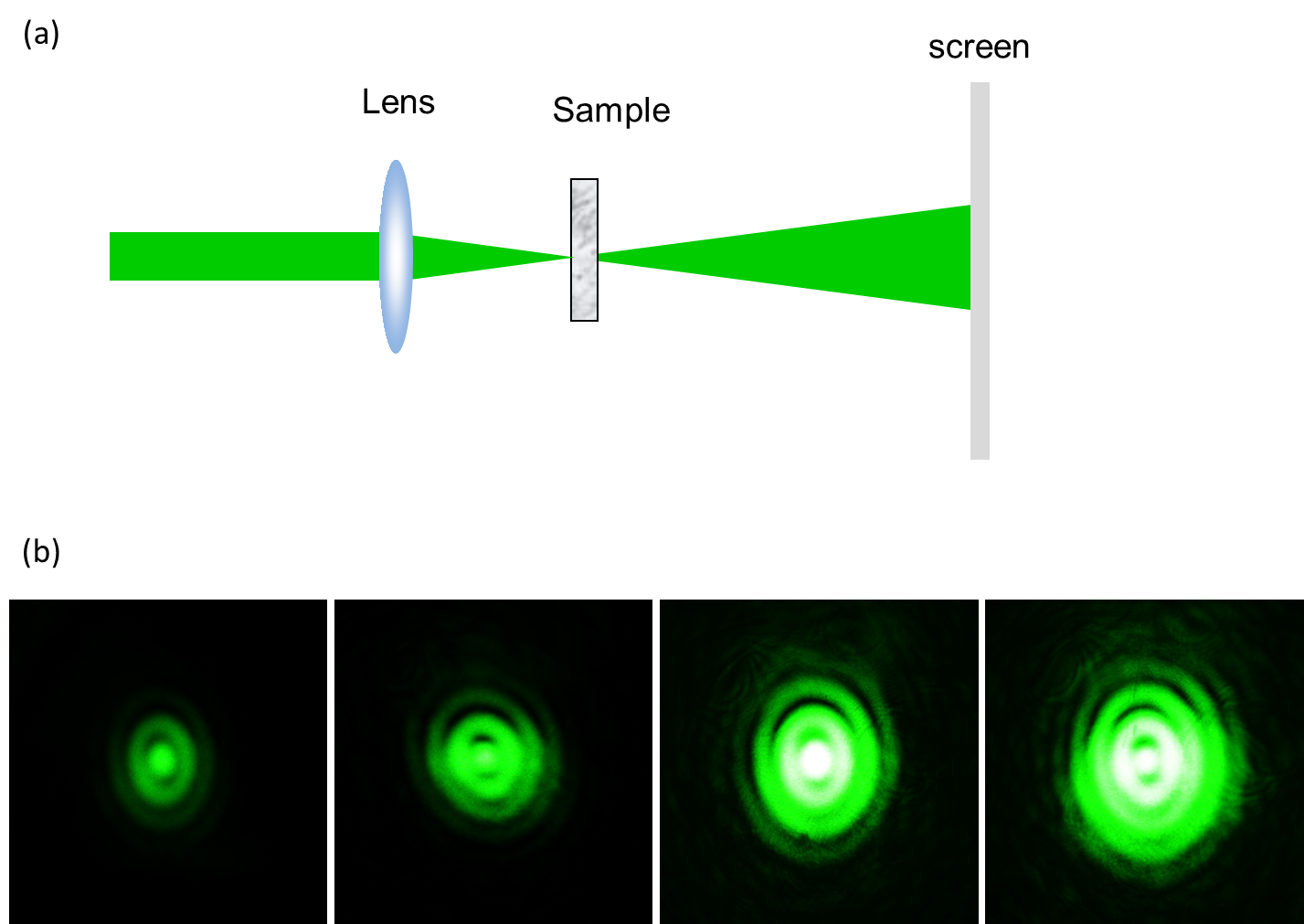


**Supporting Fig. S5** (a) Optical geometry used to record the far-field diffraction ring patterns. (b) Images of the far-field diffraction pattern of a laser beam passing through the sample at different laser powers.

On the other side, saturation absorption by the laser dye R6G has been occurred in this system. The experimental set-up is shown in Fig. S6a, a pulsed laser running at 532 nm (Continuum model PowerLite Precision 8000) horizontally passes through the R6G solution (0.8 mg/ml) and being detected by a spectrograph. As is shown in Fig. S6b, the absorption of R6G to 532 nm laser first increases with the pump power density, while keeps constant at higher pump power densities, meaning that saturation absorption is formed. In the region of saturation absorption, the effective poor cavity will become larger with increasing pump power density. While the refraction index gradient increases with increasing the pump power density. The coupling effect of the two nonlinear processes makes the effective optical path in the gain area reaching a balance state at higher pump power density.


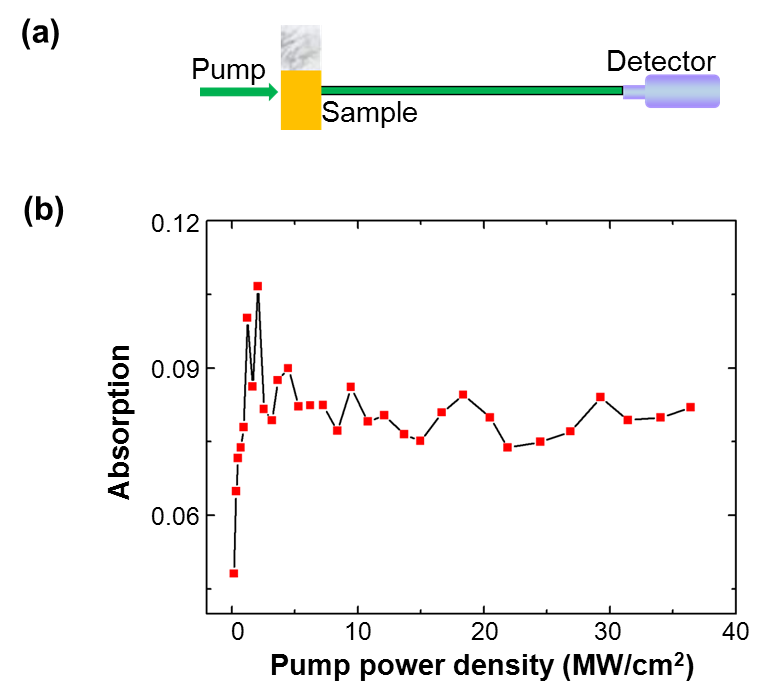


**Supporting Fig. S6** (a) Experimental set-up geometry. (b) Absorption spectrum of R6G (0.8 mg/ml) in ethanol.

Under the strong pump environment, several nonlinear optical effects (saturation absorption, large refraction index gradient for optical cavity, stimulated Raman scattering, self-phase modulation[^1-6^](#_ENREF_1), et. al.) interaction and balanced with each other, which may induce a relative stable delay time.
